# Eye-Hand Span is not an Indicator of but a Strategy for Proficient Sight-Reading in Piano Performance

**DOI:** 10.1038/s41598-019-54364-y

**Published:** 2019-11-29

**Authors:** Yeoeun Lim, Jeong Mi Park, Seung -Yeon Rhyu, Chun Kee Chung, Youn Kim, Suk Won Yi

**Affiliations:** 1 Department of Music, Graduate School, Seoul National University, Seoul, 08826 Korea; 2 Department of Transdisciplinary Studies, Seoul National University, Suwon, 16229 Korea; 3 Music and Audio Research Group, Seoul National University, Suwon, 16229 Korea; 4 Interdisciplinary Program in Neuroscience, Seoul National University, Seoul, 08826 Korea; 5 Department of Neurosurgery, College of Medicine, Seoul National University, Seoul, 08826 Korea; 6 Department of Brain and Cognitive Science, Seoul National University, Seoul, 08826 Korea; 7 Department of Music, School of Humanities, The University of Hong Kong, Pokfulam, Hong Kong; 8 Western Music Research Institute, Seoul National University, Seoul, 08826 Korea; 9 Department of Composition, College of Music, Seoul National University, Seoul, 08826 Korea

**Keywords:** Visual system, Human behaviour

## Abstract

Eye-hand span, i.e., the distance between a performer’s fixation and execution of a note, has been regarded as a decisive indicator of performers’ competence in sight-reading. However, integrated perspectives regarding the relationship between eye-hand span and sight-reading variables have been less discussed. The present study explored the process of sight-reading in terms of three domains and their interrelations. The domain indicators included musical complexity and playing tempo (musical domain), eye-hand span (cognitive domain), and performance accuracy (behavioural domain). Thirty professional pianists sight-read four musical pieces with two different complexities and playing tempi. We measured the participants’ eye-hand span, evaluated their performance accuracy, and divided the participants into three groups according to their performance accuracy values. Interestingly, we found that the eye-hand span did not change solely based on the performance accuracy. In contrast, the relationship between the eye-hand span and performance accuracy changed according to the difficulty of the sight-reading task. Our results demonstrate that the eye-hand span is not a decisive indicator of sight-reading proficiency but is a strategy that can vary according to the difficulty of sight-reading tasks. Thus, proficient sight-readers are performers who are skilled at adjusting their eye-hand span instead of always maintaining an extended span.

## Introduction

Among the various musical aptitudes, sight-reading (i.e., playing musical notation without prior rehearsal) is one of the most fundamental performance skills that all musicians should acquire^1–5^. Because performers rapidly convert symbolic musical notation into motor execution under strict temporal regulation^3^, sight-reading requires not only excellent instrumental skills but also extensive knowledge of stylistic musical features. Therefore, performers who are excellent in musical expressivity or instrumental skills may differ from performers who are excellent in sight-reading, even among professional musicians^6^. In this case, what is a ‘good sight-reader’, and how can a person be good at sight-reading? To determine the answer, a substantial volume of literature has focused on the eye-hand span (EHS), which is the distance between a performer’s fixation and execution of a note. According to a general cognitive model of sight-reading^7^, performers are able to synchronize the extracted musical information with their motor performance through a buffer. Therefore, the EHS reveals how long visual information is stored in a buffer before serving as an output via finger movements and has been used as an important indicator to explain the characteristics of working memory capacity in sight-reading proficiency^7,8^.

**Figure 1:**
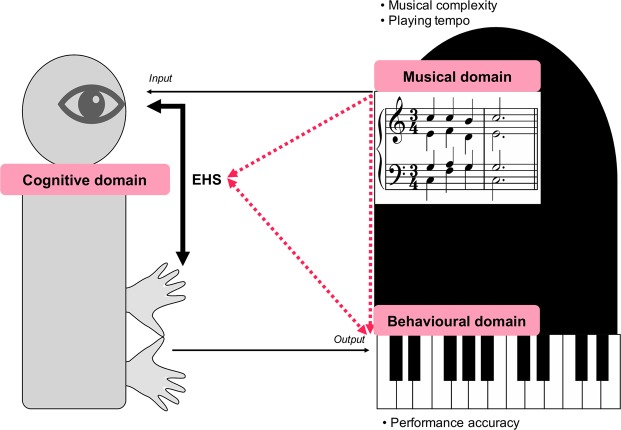
A schematic illustration of the research.

The present study explores the sight-reading process in terms of three domains (musical, cognitive, and behavioural) and investigates their interrelations. In our classification, the external visual stimuli, delivery of the encoded information from the eyes to the hands, and output of sound represent the musical, cognitive, and behavioural domains of reference, respectively (Fig. 1). The rationale underlying the investigation of the interrelationships among the domains and the variable factors is as follows.

### Relationship between the Cognitive and Behavioural Domains

In a pioneering study investigating the relationship between the EHS and sight-reading proficiency^9^, the author found that skilled sight-readers looked approximately 6.8 notes ahead of their hands, while less-skilled sight-readers looked only 3.8 notes ahead (Supplementary Table S1). Hence, the lengths of the EHS appear to differ depending on sight-reading abilities, supporting the assumption that more proficient sight-readers can more efficiently recognize and process musical patterns, thereby storing more information in a limited buffer capacity^8^.

However, a question arises when comparing two studies that directly analysed the correlation between the EHS and sight-reading proficiency^9,10^. Interestingly, the two studies showed contrasting results. The correlation between the EHS and sight-reading accuracy was strongly positive in the study conducted by Sloboda^9^ but almost absent in the study conducted by Rosemann *et al*.^10^. What is the cause of this discrepancy? Most previous studies have examined the relationship between the EHS and sight-reading proficiency only in terms of quantitative aspects of the performance (i.e., the total playing duration). Participants who played faster were regarded as skilled sight-readers, and conclusions, such as that skilled sight-readers (equal to faster players) had a longer EHS, were drawn^8,11–16^. However, as already indicated in previous studies^10,17–19^, a problem associated with uncontrolled playing tempo across participants is that the EHS is supposedly longer when playing the same length of a sight-reading piece at a faster tempo. For example, Huovinen *et al*.^18^ noted that knowledge regarding the influence of controlled playing tempo on looking ahead in sight-reading is limited because previous research concerning the EHS mostly has not externally regulated the participants’ playing tempo. Therefore, the playing tempo should be controlled across participants, particularly when investigating the relationship between the EHS and sight-reading proficiency. Only a few studies, such as those conducted by Huovinen *et al*.^18^ and Penttinen *et al*.^17^, have explored the relationship between the span measurements and sight-reading proficiency while taking into account qualitative aspects of the performance (i.e., performance accuracy) under a controlled playing tempo. Nevertheless, the relationship between the EHS and the quality of the performance not only in a single melody but also in a dual-staved musical piece remains to be investigated. In this sense, the study conducted by Rosemann *et al*.^10^ is especially noteworthy in three regards. Their experimental condition was similar to a real condition of sight-reading because the authors used dual-staved music, the authors objectively measured the EHS by making the participants play at an identical tempo to investigate the relationship between the EHS and sight-reading skills in terms of the quality of the performance, and all participants were professional pianists. The experimental results reported by Rosemann *et al*.^10^ showed no significant correlations between the EHS and sight-reading proficiency, implying that professionals’ sight-reading strategy might not be limited to looking farther ahead at musical notations (i.e., the eyes are ahead of the hands) as much as possible. Nevertheless, in the study by Rosemann *et al*.^10^, the definition and characteristics of musical complexity, which was an independent musical variable of the EHS, were not clearly presented. Moreover, only the EHS in the beat and time indices was calculated, and the EHS in the note index was excluded. To overcome the lack of information regarding the relationship between the EHS and sight-reading skills, the present study aimed to measure the EHS in the note, beat and time indices and investigate the correlations between the EHS and performance accuracy in relation to objectively and quantitatively defined musical complexity.

### Relationship between the musical and cognitive domains

An intriguing issue in the precedent literature is that the average length of the EHS converged to approximately one second in several studies that measured the EHS in the time index^8,10,11,17^, although the EHS values in the note and beat indices varied in each study. Additionally, some studies measuring the EHS in the time index showed that the temporal EHS did not change depending on sight-reading skills^8^ or the musical complexity^11^. How can these findings be interpreted? One assumption is that all sight-readers may read a certain distance of musical notation ahead regardless of how many notes or beats enter a fixed time window. In this case, the characteristic of the EHS might be time consistency. However, according to studies measuring the EHS in the note and beat indices, the EHS is affected by expertise and the complexity of the music. For instance, Huovinen *et al*.^18^ and Penttinen *et al*.^17^ found that expert music readers looked farther ahead in scores than less proficient music readers. In a conversion to absolute time measurements, Huovinen *et al*.^18^ reported an expertise effect of 300–400 ms in the median spans in favour of the more experienced of the two participant groups. Methodologically, Huovinen *et al*.^18^ redefined looking ahead as a metrical distance between fixation and a corresponding point of metrical time at the onset of fixation on the score and showed that the melodic complexity had an influence on the eye-time span (ETS). In particular, the ETS significantly changed according to the musical complexity of the sight-reading pieces. However, because no previous study has measured the EHS with all three indices simultaneously, whether the time-consistent EHS remains valid when measuring the EHS simultaneously in the note, beat, and time indices and, if not, which of the indices is a valid unit representing the EHS remain to be investigated.

### Relationship between the musical and behavioural domains

What are the musical parameters that influence performance accuracy in sight-reading? In many cases, previous studies have attempted to examine the variable of musical complexity. However, there are two limitations. First, the definition or standard of musical complexity is vague. Second, the complexity of music has not been objectively examined and has been described by uncertain musical characteristics with an ambiguous criterion. For example, the difficulty of sight-reading materials was not represented^8^ or was determined by the authors’ subjective ratings^10^. Furthermore, the sight-reading pieces were extracted or composed quite distant from actual sight-reading situations (i.e., a short single melody:^9,14,15^). Some studies used existing musical pieces to mimic the actual sight-reading condition^8,10,13,16^; however, in these studies, the definition of the musical features (i.e., complexity) was vague or the authors did not define an objective evaluation of the features^10,11,16^. In addition, the EHS has been measured under an inequitable condition in which different sight-reading pieces were assigned to different participants^8^. In a review of music-reading and eye-movement research^20^, the authors mentioned the following major deficiencies in sight-reading materials: (1) a lack of concentration on the musical structure and (2) a disconnect with general theories of music perception and cognition. The authors suggested the development of a fine-grained approach to assessing musical stimuli rather than examining the ‘coarsely defined properties’ of music, such as well-formed melodies. In this study, two levels of musical complexity will be accurately defined in terms of pitch chromaticism and the number of notes per beat. We hope that such a fine-grained approach to sight-reading materials allows for a clear exploration of the degree to which musical complexity influences performance accuracy and the length of the EHS.

## Methods

### Participants

In total, 31 students (30 females and 1 male; mean age = 21.9 years, SD = 2.0 years) majoring in piano at Seoul National University participated in this study. The participants had started learning to play the piano when they were 6.0 years old (SD = 1.0 year, range 4–7 years) and had played for 15.9 years (SD = 2.2 years, range 12–20 years) on average. The participants had accumulated substantial professional experience as they started majoring in piano as their primary instrument at an average age of 11.4 years (SD = 2.3 years, range 6–16 years) and had majored in piano for 10.5 years (SD = 2.9 years, range 5–18 years). Based on the accumulated time the participants had spent expertly playing the piano, we determined that they should all be considered professional pianists in terms of general piano skills and that there were no novices or intermediate pianists among the participants. All participants were right-handed and had normal or corrected-to-normal vision. All experimental protocols were approved by the Institutional Review Board (IRB) of Seoul National University (1805/003-017). All methods were carried out in accordance with the relevant guidelines and regulations. Written informed consent was obtained from all subjects. The study was conducted in accordance with the ethical standards outlined in the 1964 Declaration of Helsinki.

### Sight-reading materials

#### Standards of musical complexity

Musical complexity was rigorously defined based on pitch chromaticism and the number of notes per beat to reflect both the pitch and temporal aspects of the music. Because pitch and rhythm have been regarded as fundamental elements in the history of Western classical music, this study used these two musical elements as the determinants of musical complexity. For example, the more chromatic (i.e., non-diatonic notes), the higher the musical complexity level. Similarly, the more notes per beat, the higher the musical complexity level.

#### Composition

**Figure 2:**
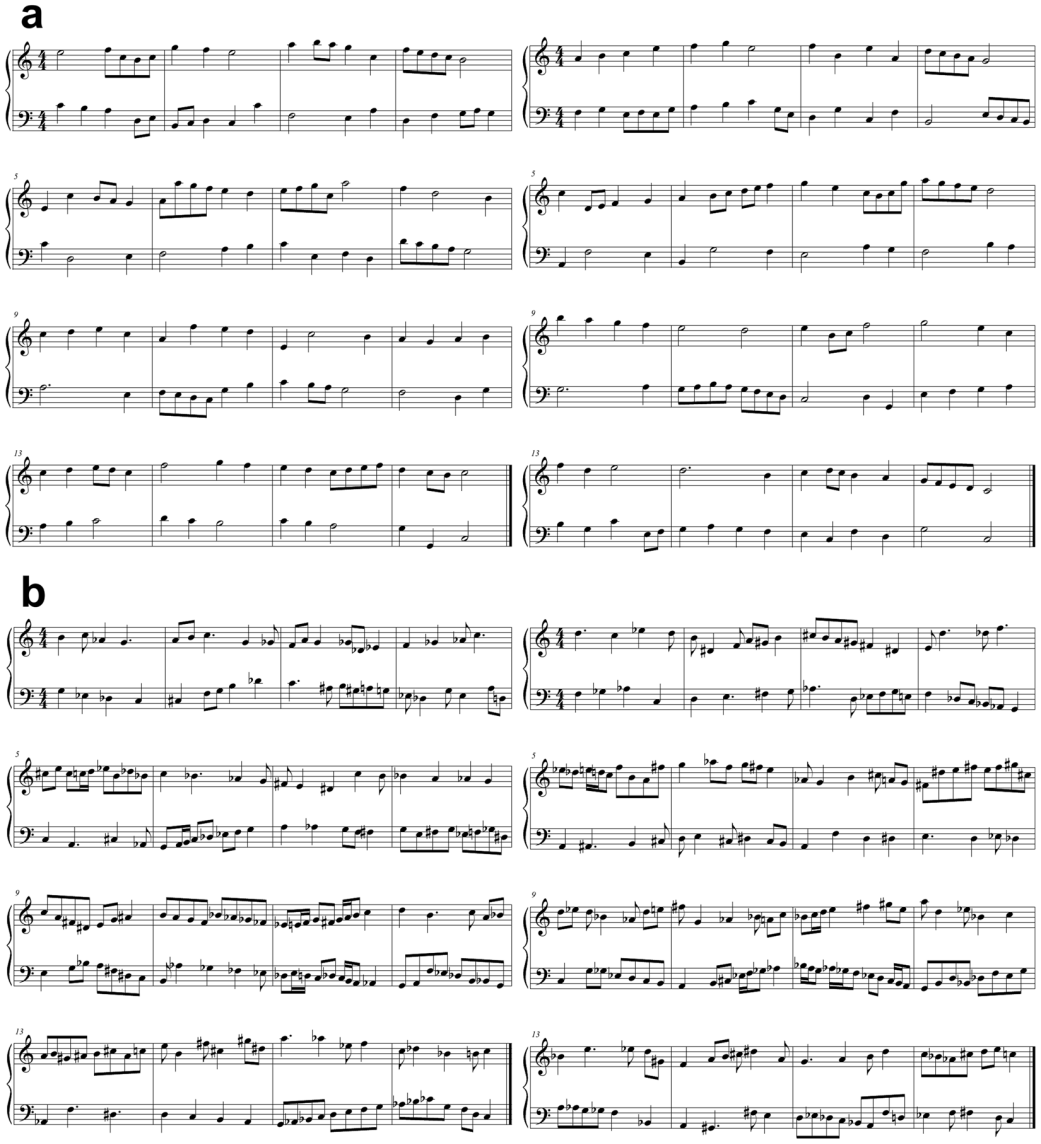
(**a**) Simple pieces 1 (left) and 2 (right). **(b)** Complex pieces 1 (left) and 2 (right).

Four musical pieces with two different complexities (simple and complex) were composed specifically for this study. The level of complexity was objectively differentiated by quantitatively comparing the sight-reading materials. As shown in Supplementary Table S8, the simple and complex pieces had different numbers of accidentals, notes per beat, and total number of notes. In addition, there were no rests in any piece. To exclude the effect of repetition, each level of complexity had two different pieces (with quantitatively similar musical components). For instance, any two pieces with the same level of complexity possessed the same average number of notes per measure. All pieces had the same key signature (C Major), time signature (4/4 metre) and length (16 measures) to prevent the introduction of confounding variables other than the complexity and playing tempo. Figure. 2a,b provide examples of the simple and complex pieces, respectively.

#### Measurement of the musical complexity

The degree of complexity was investigated to demonstrate the nature of the sight-reading materials. Specifically, the entropy of the sight-reading materials was calculated, and the entropy value was compared to the entropy value of representative composers from different periods^21,22^. Entropy is derived from information theory and is a mathematical tool used to measure the value of information^23^. Entropy H is determined by the following formula:

1 $${\rm{{\rm H}}}({\rm{{\rm X}}})=-\,\mathop{\sum }\limits_{i=1}^{n}(\,\,{P}_{i}{\log }_{2}{P}_{i})$$

where H(X) is the entropy of information X and *P*_*i*_ is the probability of an event occurring with character number *i* appearing in a stream of characters in Eq. (1). Entropy increases as the number of possible outcomes increases and the probability of each outcome becomes equivalent.

**Figure 3:**
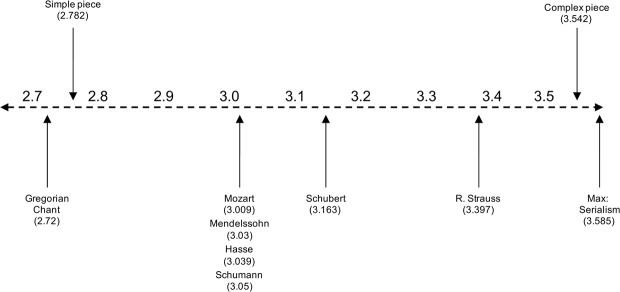
Comparison of the degree of chromaticism between the sight-reading materials and different styles of Western music. The small vertical arrows below the horizontal dashed line indicate the entropy value of each composer based on the references. For the references, we used the entropy values of a Gregorian chant, Mendelssohn and Schumann from Youngblood^22^, and Mozart, Hasse, and R. Strauss from Knopoff and Hutchinson^21^. The two vertical arrows above the dashed line indicate the entropy values of the sight-reading materials used in the present experiment.

In music research, the number of possible outcomes is often considered the number of pitch classes^21,22,24–26^, with a maximum of 12 probabilities. Entropy is higher when the 12 notes appear at an equal frequency. The music of the 12-tone technique in which all 12 notes are given equal importance is the most complex type of music. Therefore, the entropy of music represents the degree to which a piece is chromatic. To demonstrate the degree of chromaticism in the sight-reading materials, the entropy of the materials was calculated and compared to several references from Youngblood^22^ and Knopoff and Hutchinson^21^. Figure 3 shows a comparison of the entropies between the sight-reading materials and references with different styles of music. In Fig. 3, entropy is shown to gradually increase throughout the history of Western music from the Gregorian chants in the Middle Ages to the music of the 20^th^ century. The simple piece was located between the period of the Middle Ages and the Classical period, and the complex piece had an entropy value nearly equivalent to 12-tone music. This comparison can be used to estimate how chromatic the sight-reading materials are compared to the works of Western classical composers.

### Equipment

The sight-reading materials were presented on a 23″ monitor with a resolution of 1920 × 1080 pixels. Binocular movements were recorded using Tobii Pro Glasses 2 (Tobii Technology, Stockholm, Sweden) at a sampling rate of 50 Hz (every 20 milliseconds). The distance between the eye and the monitor was 50 cm. The participants were required to stabilize their heads as much as possible but were also able to glance at their fingers to maintain a natural condition during sight-reading. A Yamaha CLP-525 Clavinova digital piano was used in the experiment, and the participants’ performances were directly recorded by Logic Pro X 10.2.2 in MIDI format.

### Procedures

There were two contrasting tempi per level of complexity. All participants played four sight-reading pieces in two different tempi (simple, slow; simple, fast; complex, slow; and complex, fast). The playing tempi of the two pieces of a given complexity were counterbalanced. Specifically, each piece of a given complexity could be performed at both slow and fast playing tempi and was randomly assigned to either a slow or fast tempo per participant. For example, if a participant played one of the two pieces of a given complexity at a fast tempo, the participant then played the other piece of the two pieces of the given complexity at a slow tempo and vice versa. The playing tempi were 80 BPM under the slow tempo condition and 104 BPM under the fast tempo condition. The pieces were organized into eight different presentation orders. Within the succession of the sight-reading pieces, the level of complexity did not gradually increase or decrease, and playing tempo did not become gradually slower or faster. Similar to Huovinen *et al*.^18^’s method of randomizing presentation orders, the assignment of the participants to one of the eight orders of presentation was randomized by allowing them to select an experimental schedule and by rotating the order of presentation between every successive participant. The participants were instructed to perform the given sight-reading materials accurately with regard to pitch and rhythm, excluding any musical expression or interpretative elements such as timing, dynamics, or articulation. Before starting each session, the eye tracker was calibrated at four different points on the sheet music. The participants fixated at each calibration point for at least three seconds. After the calibration phase, a metronome was provided for two measures before playing; then, the participants started to sight-read along with the metronome. The metronome was provided for each beat to help the participants maintain a constant tempo. In total, the experiment lasted approximately 20 minutes. After the main experiment, the participants were asked to complete a short questionnaire about their musical experience.

### Data analysis

#### Eye movement

Using MATLAB-based programs, the EHS was calculated as the (1) latency (in ms) between fixating and playing a note, (2) number of beats and (3) notes between the current eye and hand position. The EHS was computed for each beat of each measure, ultimately yielding 48 data points. The beat and time spans were proportional to each other because the time span was calculated as the beat span multiplied by the duration of a beat in a given tempo. Further details of the data analysis of the eye movements are provided in the Supplementary Information.

#### Performance accuracy

For the evaluation of performance accuracy, we analysed the integrated accuracy, including pitch and temporal information, by comparing the participants’ performances with deadpan MIDI performances of the same pieces. Additionally, we counted the number of pitch and inter-onset-interval (IOI) errors to demonstrate the accuracy of the pitch and rhythmic aspects separately. Further details of the measurements of performance accuracy are provided in the Supplementary Information.

## Results

### Performance accuracy depending on the musical complexity and playing tempo

**Figure 4:**
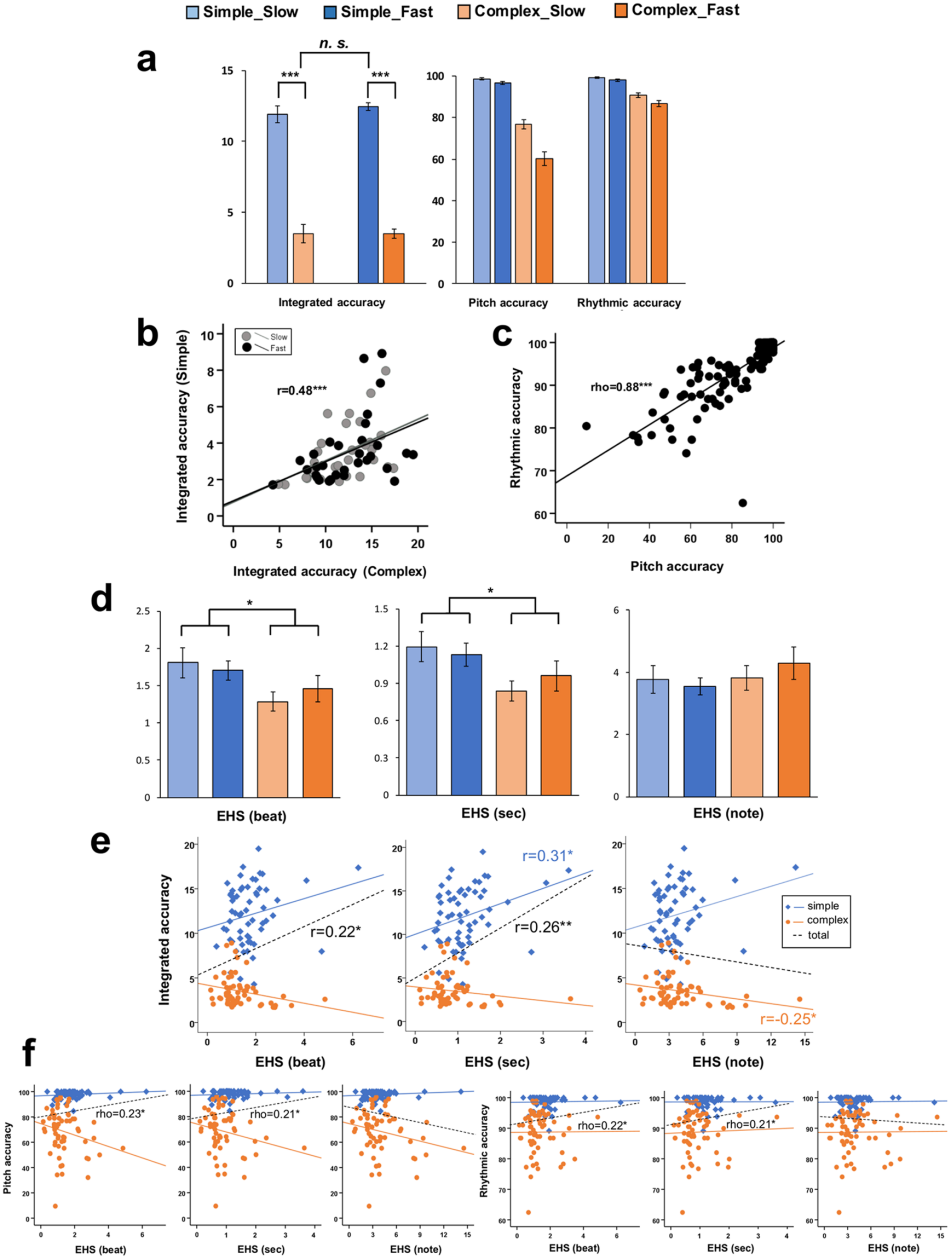
(**a**) The integrated, pitch, and rhythmic accuracy for different sight-reading tasks (simple-slow, simple-fast, complex-slow, and complex-fast). (**b**) Scatter plot depicting the regression of the correlation between the performances of the simple and complex pieces in the slow and fast tempi in terms of the integrated accuracy. (**c**) Scatter plot depicting the regression of the correlation between pitch and rhythmic accuracy. (**d**) The EHS (beat, sec, and note) values for different sight-reading tasks (simple-slow, simple-fast, complex-slow, and complex-fast). (**e**) Scatter plot depicting the regression of the correlation between the EHS (beat, sec, and note) and integrated accuracy. (**f**) Scatter plot depicting the regression of the correlation between the EHS (beat, sec, and note) and pitch accuracy and the correlation between the EHS (beat, sec, and note) and rhythmic accuracy. ***P < 0.001, **P < 0.01, *P < 0.05.

The integrated, pitch, and rhythmic accuracy depending on the four types of sight-reading tasks (simple-slow, simple-fast, complex-slow, and complex-fast) were assessed with a repeated-measures two-way analysis of variance (ANOVA) with musical complexity and playing tempo as factors. Regarding the integrated accuracy, we found that the accuracy values for the simple piece were significantly higher than those for the complex piece [*F* (1, 30) = 314.86, *P* < 0.001] (Supplementary Tables S2 and S3 and Fig. 4a). However, there was no significant difference due to playing tempo [*F* (1, 30) = 2.38, *P* = 0.461], and there was no interaction effect (complexity × tempo) [*F* (1, 30) = 1.80, *P* = 0.823]. Regarding the pitch and rhythmic accuracy, we found that the accuracy values for the simple piece were significantly higher than those for the complex piece [*F* (1, 30) = 149.16, *P* < 0.001; *F* (1, 30) = 112.95, *P* < 0.001] and that the accuracy values for the slow piece were significantly higher than those for the fast piece [*F* (1, 30) = 68.89, *P* < 0.001; *F* (1, 30) = 16.31, *P* < 0.001]. We also found an interaction effect (complexity × tempo) [*F* (1, 30) = 54.04, *P* < 0.001; *F* (1, 30) = 4.36, *P* = 0.045]. As shown in Fig. 4a, the interaction effect suggests that playing tempo had a greater influence on performance accuracy for the complex piece than for the simple piece. To investigate the influence of the four types of sight-reading tasks (simple-slow, simple-fast, complex-slow, and complex-fast) on pitch and rhythmic accuracy, we also conducted a repeated-measures one-way ANOVA with the difficulty of the sight-reading tasks as a factor. According to the results, the pitch and rhythmic accuracy differed significantly depending on the difficulty of the sight-reading tasks [*F* (3, 90) = 125.16, *P* < 0.001; *F* (3, 90) = 62.06, *P* < 0.001]. Using Bonferroni correction, we found that the higher the difficulty of the sight-reading tasks, the lower the pitch and rhythmic accuracy (simple-slow > simple-fast > complex-slow > complex-fast; *Ps* ≤ 0.002; *Ps* ≤ 0.02).

Comparing the integrated accuracies measured for the participants’ pairs of performances in a given tempo, we found a significant positive Pearson correlation between the measurements for the two levels of complexity (*r* = 0.48, *P* < 0.001; Fig. 4b). These results suggest that the participants who played the simple piece in a given tempo relatively accurately also played the complex piece in this tempo with high accuracy. To investigate the correlation between the pitch and rhythmic accuracy, a Spearman correlation coefficient analysis was conducted. As shown in Fig. 4c, we found a significant positive correlation between the pitch and rhythmic accuracy (rho = 0.88, *P* < 0.001).

### EHS depending on the musical complexity and playing tempo

The EHS values depending on the four types of sight-reading tasks (simple-slow, simple-fast, complex-slow, and complex-fast) were also assessed with a repeated-measures two-way ANOVA with musical complexity and playing tempo as factors. Supplementary Table S4 shows the three types (beat, sec, and note) of EHS values depending on the complexity and playing tempo. We found a main effect of complexity, but not playing tempo, on the EHS (beat) and EHS (sec) but not EHS (note). The EHS (beat and sec) values for the simple piece were greater than those for the complex piece [*F* (1, 30) = 6.39, *P* = 0.017; *F* (1, 30) = 7.12, *P* = 0.012]. However, there was no significant difference due to playing tempo [*F* (1, 30) = 0.06, *P* = 0.802; *F* (1, 30) = 0.11, *P* = 0.741], and there was no interaction effect (complexity × tempo) [*F* (1, 30) = 0.64, *P* = 0.431; *F* (1, 30) = 0.68, *P* = 0.416] (Supplementary Table S5 and Fig. 4d). To investigate the influence of the four types of sight-reading tasks (simple-slow, simple-fast, complex-slow, and complex-fast) on the EHS (beat, sec, and note), we also conducted a repeated-measures one-way ANOVA with the difficulty of the sight-reading tasks as a factor. According to the results, the EHS (beat, sec, and note) values showed no significant difference depending on the difficulty of the sight-reading tasks [*F* (3, 90) = 2.36, *P* = 0.077; *F* (3, 90) = 2.597, *P* = 0.057; *F* (3, 90) = 3.029, *P* = 0.605].

### Correlations between the EHS and performance accuracy

We conducted a Pearson correlation coefficient analysis of the correlation between the EHS and integrated accuracy and Spearman coefficient analyses of the correlation between the EHS and the pitch and rhythmic accuracy. Overall, we found a significant positive correlation between the EHS and integrated accuracy in the indices of beat (*r* = 0.22, *P* = 0.016) and sec (*r* = 0.26, *P* = 0.004; Fig. 4e). Additionally, as shown in Fig. 4f, a significant positive correlation was found between the EHS and the pitch and rhythmic accuracy in the indices of beat (rho = 0.23, *P* = 0.01; rho = 0.22, *P* = 0.014) and sec (rho = 0.21, *P* = 0.022; rho = 0.21, *P* = 0.025). However, as shown in Supplementary Table S6 and Fig. 4e,f, we found different correlation tendencies depending on the musical complexity for the integrated and pitch accuracy. The EHS values for the simple piece tended to be positively correlated with the integrated accuracy [*r* = 0.31, *P* = 0.015 (sec)], whereas the EHS values for the complex piece tended to be negatively correlated with the integrated accuracy [*r* = −0.25, *P* = 0.049 (note)]. Regarding pitch accuracy, the differences in the correlation tendencies depending on the musical complexity were not significant; however, the contrasting correlation tendencies depending on the musical complexity suggested that the participants might have used a different strategy depending on the musical complexity because the performer’s perceptual difficulty in sight-reading tasks can vary. Therefore, we divided the participants into groups according to their performance accuracy values and conducted a correlation analysis of the EHS and performance accuracy according to the difficulty of the sight-reading tasks per group.

### Correlations between the EHS and performance accuracy according to the difficulty of the sight-reading tasks in the high and low groups

We divided the participants into three groups (*n* = 10, 11, and 10) according to their performance accuracy values of the four types of sight-reading tasks (simple-slow, simple-fast, complex-slow, and complex-fast). To investigate the different correlation tendencies of the EHS and performance accuracy depending on the difficulty of the sight-reading tasks, we conducted Spearman correlation coefficient analyses between the EHS (beat, sec, and note) values and performance accuracy (integrated accuracy, pitch accuracy, and rhythmic accuracy) values per group. To clarify the differences in the groups, we conducted statistical analyses of 10 subjects with the highest performance accuracy and 10 subjects with the lowest performance accuracy on each task.

**Figure 5:**
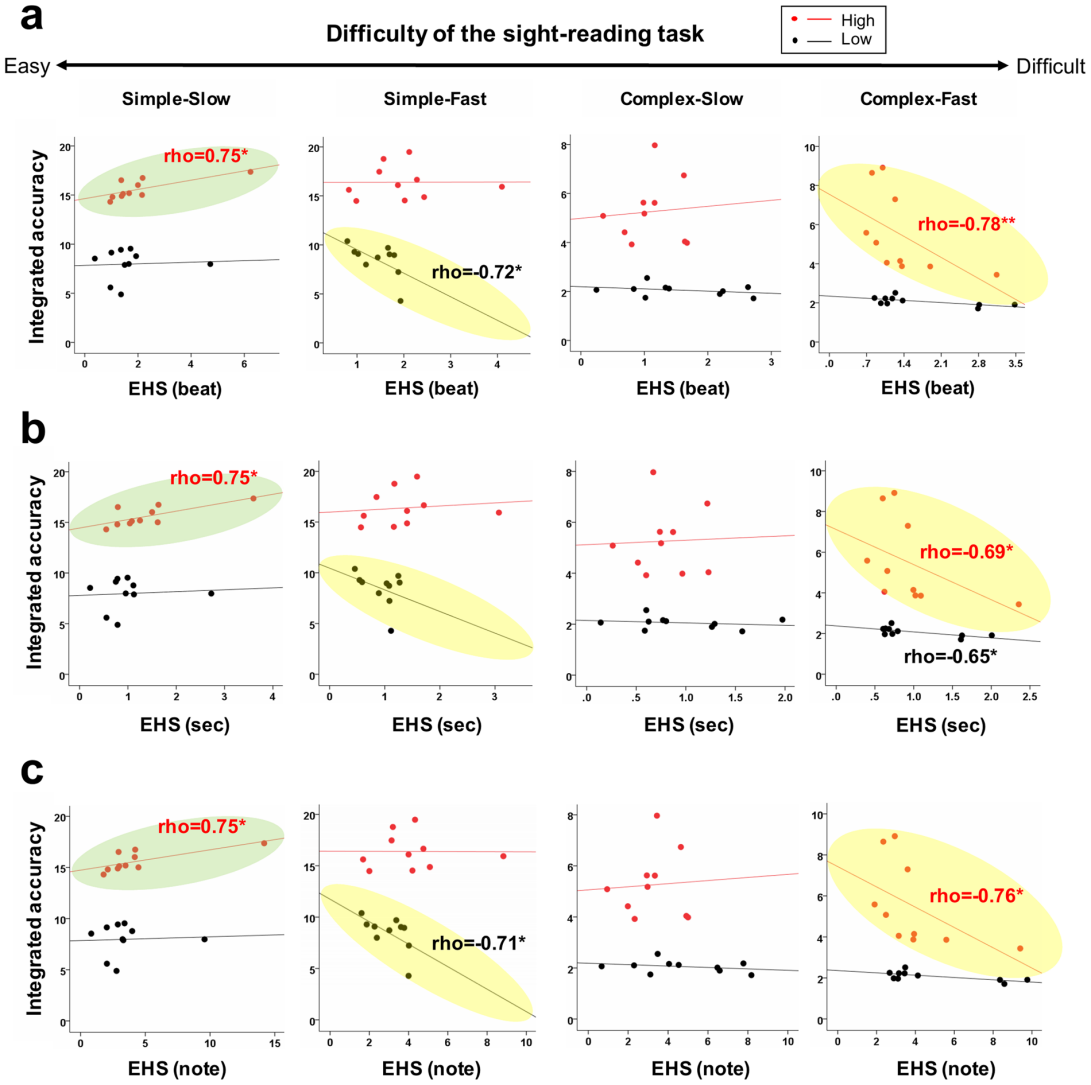
Correlations between the EHS (beat, sec, and note) and integrated accuracy for different difficulties of the sight-reading task in the high and low groups. (**a**) Correlation between the EHS (beat) and integrated accuracy. (**b**) Correlation between the EHS (sec) and integrated accuracy. (**c**) Correlation between the EHS (note) and integrated accuracy. **P < 0.01, *P < 0.05.

As shown in Supplementary Table S7 and Fig. 5, the correlation tendency between the EHS and integrated accuracy differed according to the difficulty of the sight-reading task. Although there were several outliers, they were treated as a rank using nonparametric analyses. In the high group, a positive correlation was found between the EHS (beat, sec, and note) and the integrated accuracy in the easiest task (simple-slow) [Spearman’s rho = 0.75, *P* = 0.013], while a negative correlation was found in the most difficult task (complex-fast) [rho = −0.78, *P* = 0.008 (beat), rho = −0.69, *P* = 0.029 (sec), rho = −0.76, *P* = 0.011 (note)]. However, in the low group, a negative correlation was found overall. In particular, a significant negative correlation was observed in the simple-fast task [rho = −0.72, *P* = 0.019 (beat), rho = −0.71, *P* = 0.022 (note)]. Regarding pitch and rhythmic accuracy, we found no significant correlations of the EHS with pitch and rhythmic accuracy depending on the difficulty of the sight-reading tasks.

## Discussion

The most intriguing finding of the present study is that the relationship between the EHS and performance accuracy varied with the difficulty of the sight-reading tasks. Specifically, by dividing the performer groups based on their performance accuracy values for the four types of sight-reading tasks (simple-slow, simple-fast, complex-slow, and complex-fast), we found differing correlation tendencies between the EHS and performance accuracy under the two complexity conditions. In the high-performance group, a positive correlation was found between the EHS (beat, sec, and note) and the integrated accuracy in the easiest task (simple-slow), whereas a negative correlation was found in the most difficult task (complex-fast). In contrast, in the low-performance group, a negative correlation was found overall in the sight-reading tasks. In particular, a significant negative correlation was observed in the simple-fast task. This result suggests that it is difficult to determine sight-reading proficiency based on the EHS alone. While our findings are compatible with Huovinen *et al*.^18^ and Penttinen *et al*.^17^, who found that proficient music readers looked farther ahead in scores than less proficient music readers, our results thus also suggest that the EHS does not seem to be a decisive indicator of sight-reading proficiency. We assume that a proficient sight-reading performer does not always have a longer EHS; similarly, a less proficient sight-reading performer does not always have a shorter EHS. In contrast, we suggest that the relationship between the EHS and performance accuracy might be significantly associated with the difficulty of the sight-reading tasks. Specifically, participants with better-than-average sight-reading skill (skill involved in decoding and converting the visual symbols into appropriate motor movements) tend to perform more accurately and have the capacity to look farther ahead at will (resulting in a larger EHS) in a relatively easy sight-reading task. We assume that these different correlations of the relationship between the EHS and performance accuracy depending on sight-reading proficiency might be due to varying degrees of the performer’s perceptual difficulty in sight-reading tasks. Thus, although the combination of musical complexity and playing tempo determined the objective difficulty of the sight-reading tasks, the perceptual difficulty might have differed by sight-reading proficiency, resulting in varying correlations between the EHS and performance accuracy. Therefore, sight-reading proficiency can be explained by examining the EHS in terms of multidimensional rather than unidimensional domains of reference, including musical domains.

Few studies regarding the visual processes in music reading have focused on the relationship between the musical, cognitive, and behavioural domains. For example, Huovinen *et al*.^18^ explored how local changes in the musical stimulus influenced the ETS and found that the ETS was significantly affected by local melodic complexity while sight-reading single-line melodies. In addition to the need to investigate similar complexity effects in the context of piano music written on two staves, the starting point of the present article was a discrepancy in previous studies regarding the relationship between the EHS and performance accuracy. In particular, whereas Sloboda^9^ reported a strong positive correlation between the EHS and performance accuracy, no similar correlation was observed by Rosemann *et al*.^10^. Our study suggests an explanation regarding the relationship between the EHS (cognitive domain) and performance accuracy (behavioural domain) by taking into account the difficulty of sight-reading tasks (musical domain). If the EHS is a changeable strategy according to the difficulty of sight-reading tasks, our experimental results can be brought in line with both of the studies mentioned.

The reason for the opposite correlations reported by Sloboda^9^ and Rosemann *et al*.^10^ might be because the difficulty of their sight-reading tasks differed. In the study by Sloboda^9^, the stimuli corresponded to the easy level of difficulty in the present study, and consequently, a highly positive correlation was found between the EHS and performance accuracy. In contrast, because the sight-reading material used by Rosemann *et al*.^10^ was situated between the easy and difficult levels of our sight-reading task difficulty, a significant positive correlation was not observed between the EHS and performance accuracy. Surprisingly, according to Penttinen *et al*.^17^, the EHS seemed to be shorter more often among more experienced performers when they played altered melodies. Furthermore, Huovinen *et al*.^18^ showed that the span of looking ahead in the score may be highly sensitive to the content of the musical stimuli. By linking to the conjecture proposed by Huovinen *et al*.^18^ and Penttinen *et al*.^17^, our experimental results provide empirical evidence supporting the importance of the flexibility of the EHS as a sight-reading strategy, suggesting that proficient sight-readers seem to be more skilled in regulating their EHS than those who consistently maintain an extended EHS.

This study investigated the effect of musical complexity and playing tempo on the EHS. We found that the musical complexity significantly influenced the EHS. Interestingly, the value of the EHS (beat and sec) when playing the complex piece was demonstrated to be smaller than that when playing the simple piece; however, the value of the EHS (note) did not change depending on the musical complexity. Additionally, our results indicated that the EHS (beat and sec) did not change significantly according to the playing tempo (whether playing slower or faster), while several studies have shown the significant effect of the playing tempo on the EHS in the time index^8,11^. One striking observation is that the influence of the musical complexity on the EHS has been shown only for a beat index^10,16,18^ or a note index^11,16^, and the EHS in the time index did not change according to the musical complexity in previous studies^8,10,11^. However, in this study, both the EHS (beat) and EHS (sec) were affected by the musical complexity, and no significant difference depending on the musical complexity was found in the EHS (note). Thus, our results suggest that musical complexity can change the efficiency of processing musical notations in a limited buffer capacity rather than confirming the existence of a consistent time lag between the eyes and hands. This conclusion is consistent with the findings reported by Huovinen *et al*.^18^ that the complexity of the upcoming symbols of a score affects saccadic processes, although the tendency of the influence of the musical complexity differed between Huovinen *et al*.^18^ and the present study: the length of the span became shorter when performers were presented with the complex sight-reading material in the present study, whereas the length of the span lengthened according to the musical complexity in the study by Huovinen *et al*.^18^. The contrasting tendency in the effect of musical complexity on the span might be due to the different types of approaches used for the span measurements^18^. In Huovinen *et al*.^18^, musical complexity was always some local phenomenon (a large intervallic leap), and the authors were interested in investigating whether the span is locally adjusted in such locations (by “early attraction”). However, in the present study, whole pieces of music were more complex than others, and the measurements were related to the overall differences in span measurements in such contexts. Hence, it seems that the results of both the study by Huovinen *et al*.^18^ and the present study could be true simultaneously: (1) compared to simple pieces, in more complex pieces, musicians use somewhat shorter spans in general, and (2) when there are local differences in musical complexity, more complex elements may attract the reader’s eyes earlier, yielding locally longer spans.

The present study demonstrated the influence of musical complexity and playing tempo on performance accuracy. We found that performance accuracy was significantly affected by the musical complexity but did not vary with the playing tempo. Our results provide more concrete evidence of the influence of musical complexity. Although previous literature concerning the EHS has shown that musical complexity significantly affects sight-reading performance^11,13,16^, knowledge regarding the type of complexity and extent to which complexity has an effect on sight-reading performance is limited. To address the limitation concerning this issue, this study demonstrated the objective degree of complexity by comparing the entropy of the sight-reading pieces and references. Using a quantitative approach to measure the qualitative features of musical complexity, we were able to estimate a more definite threshold and its degree in discussing the influence of musical complexity on sight-reading and the EHS.

As might be expected, we found a strong positive correlation between participants’ performance accuracy measures in the simple and complex pieces. The participants who played the simple piece more accurately also played the complex piece at a higher accuracy. Our results imply that proficient sight-readers are performers who are not deterred by the difficulty of a sight-reading piece. If this is true, who were the proficient sight-readers, and how did they perform so well? Although these questions lie beyond the purview of the present study, one possible explanation can be discovered from previous research. Some studies have emphasized the importance of both practice-dependent and practice-independent factors on sight-reading proficiency. For example, regarding practice-dependent factors, it has been demonstrated that sight-reading achievement is strongly associated with deliberate practice^27,28^, auditory imagery skills^29–31^, the ability to improvise, and music knowledge^32^. Regarding practice-independent factors, working memory capacity^33,34^, sensorimotor speed^1,2^, intrinsic motivation^35^, unique types of representations^36^, and verbal memory^37^ have been shown to be associated with sight-reading achievement. In particular, Kopiez and Lee^1^ demonstrated that the combination of predictor variables of sight-reading proficiency varied with the level of musical complexity. These authors concluded that sight-reading proficiency can be determined by a combination of practice-dependent factors and practice-independent predictors. Linking this study to our experimental results, it seems that practice-independent and practice-dependent skills are proportional or at least positively related to one another because the performers who played the simple piece accurately also played the complex piece accurately. However, this is only a potential hypothesis without any empirical evidence. Meticulous research exploring this assumption should be undertaken in the future.

In conclusion, the present study explored the interrelationships among the three domains of the sight-reading process, namely, musical complexity and playing tempo (musical domain), eye-hand span (cognitive domain), and performance accuracy (behavioural domain). The findings concerning the varying correlations between the eye-hand span and performance accuracy depending on the difficulty of sight-reading tasks suggest that (1) the eye-hand span is not a decisive indicator of sight-reading proficiency but is a strategy that can be changed according to the difficulty of the sight-reading task, and (2) proficient sight-readers are performers who are skilled in adjusting their eye-hand span instead of always maintaining an extended span.

## Supplementary information

Supplementary Information

## Data Availability

The datasets generated and/or analysed during the current study are available from the corresponding author upon reasonable request.
