# Supplementary material for: Eye-Hand Span is not an Indicator of but a Strategy for Proficient Sight-Reading in Piano Performance
Source: Sci Rep. 2019 Nov 29;9:17906. doi: 10.1038/s41598-019-54364-y (PMC6884463; doi:10.1038/s41598-019-54364-y)
Supplement: Supplementary file 1 — Supplementary Information [file 41598_2019_54364_MOESM1_ESM.pdf]

Supplementary information for

Eye-Hand Span is not an Indicator of but a Strategy for Proficient Sight-Reading in Piano Performance

Yeoeun Lim, Jeong Mi Park, Seung-Yeon Rhyu, Chun Kee Chung, Youn Kim, and Suk Won Yi

Supplementary methods

Data analysis

Eye movement

Eye movements were recorded with an eye tracking program (Tobii Glasses Controller by Tobii Technology, Stockholm, Sweden) in real time. After the data collection process, the participants' eye movements were automatically mapped to each corresponding sight-reading score through the eye tracking analysis software (Tobii Pro Lab by Tobii Technology, Stockholm, Sweden). For each piece, we created specific events and marked the timestamp of the onset and offset of the calibration and sight-reading performance in the raw data. Only the eye movement intervals between the point of onset and offset were used in the calculation of the EHS. We used a Tobii Velocity-Threshold Identification filter (a 75-ms maximum gap length for interpolation, a maximum time/angle between fixations for merging adjacent at 75 ms/0.5 degrees, and fixations below 60-ms duration discarded–see Olsen[1] for further details and rationales for these parameters) to filter and analyse the fixations and saccades. We set the onset of the fixation as the standard of an eye to calculate the length of the EHS. The analysed eye movement data were exported into a TSV format, and the subsequent calculation and visualization were performed in the MATLAB program. The EHS was measured during the four sight-reading pieces. In total, there were 64 data points per piece because each sight-reading piece consisted of 64 beats. The length of the EHS was calculated per beat, and the representative EHS per piece was calculated as the mean value of the sum of the

values of all beats divided by 64. Specifically, we calculated the numbers of notes and beats that occurred between the onset of fixation and performance as a discrete event and a time delay between the two onsets of a note (latency: sec). The average EHS was obtained by adding all values and dividing by 64 per index. The beat and time spans were proportional to one another because the time span was the beat span multiplied by the playing tempo. For instance, in Supplementary Fig. S1 online, the green circles indicate the fixation orders that correspond to each beat, which is represented in the upper side of the rectangle. Thus, the tenth fixation is located in the first note of beat 12, and the value of the EHS at this point is three notes or two beats.

Performance accuracy

For the evaluation of the performance accuracy, we analysed the integrated accuracy, pitch accuracy, and rhythmic accuracy. First, the integrated accuracy was analysed by the dynamic time warping (DTW) algorithm, which compares two different sequential datasets that vary in time and speed[2,3]. In music research, the DTW algorithm has been utilized for the assessment of music performance as a quantitative measurement of the performance accuracy[4-7]. Because this algorithm measures the similarity between the performance and reference by a frame unit (10 ms) rather than a note unit, it is possible to detect all subtle differences between the two performances through the algorithm. In the present study, a deadpan MIDI, which did not contain any types of variation in dynamic, tempo or articulation, was used for the reference performance because the participants were instructed to perform the given sight-reading materials accurately only in pitch and rhythm, excluding any musical expression or interpretative elements, such as timing, dynamic, or articulations. Using the algorithm, we calculated

the overall similarities in pitch and temporal information between each performance and the reference MIDIs. To evaluate the performance accuracy not only synthetically but also separately, we analysed the accuracy of pitch and rhythm. For the pitch accuracy, we counted the number of pitch errors per performance dataset. In contrast to a single melody in which pitch errors can be manually and relatively easily counted, we had to use a method to automatically count the pitch errors because it was difficult to manually count the pitch errors in our performance data; the sight-reading materials used in the present study were polyphonic-style pieces, and there were over 124 performance MIDI files (31 participants performed four sight-reading pieces). To automatically count the pitch errors in each participant's performance, we applied the method described by Nakamura *et al.*[8], which is a state-of-the-art MIDI-to-MIDI alignment method provided as an open source. While counting the pitch errors, "missed notes", "added notes", and "incorrect notes" were regarded as types of pitch errors with reference to Huovinen *et al.*[9]. In our study, "missed notes" indicate notes that existed in the score but were not hit in the performance, "added notes" indicate notes that did not exist in the score but were hit in the performance, and "incorrect notes" indicate notes that were hit in the performance but were incorrect pitches. The value of the pitch accuracy was derived by dividing the number of pitch errors ($\#\ of\ missed\ notes + \#\ of\ added\ notes + \#\ of\ pitch\ errors$) by the number of notes available in the piece (simple: 130 notes and complex: 190 notes) and multiplying by 100 to represent the pitch accuracy more intuitively. For the rhythmic accuracy, we also simply counted the temporal errors similarly to the calculation of the pitch accuracy. We calculated the inter-onset-interval (IOI) of each reference and performance MIDI and counted the number of "wrong IOIs"

in the performance MIDI compared to the corresponding reference MIDI. Each IOI was determined to be right or wrong based on a certain threshold. If an IOI deviation was larger than the threshold, the IOI was counted as a wrong hit, indicating a temporal error. We set the minimum threshold as a length of the 32nd note considering that the shortest note in the four sight-reading materials was the 16th note and that the shortest note varies in time (ms) depending on the playing tempo (slow or fast). Similar to the method used to obtain the pitch accuracy, the number of incorrect IOIs was divided by the number of IOIs in the piece (simple: 129 IOIs and complex: 189 IOIs) and multiplied by 100.

Supplementary figures

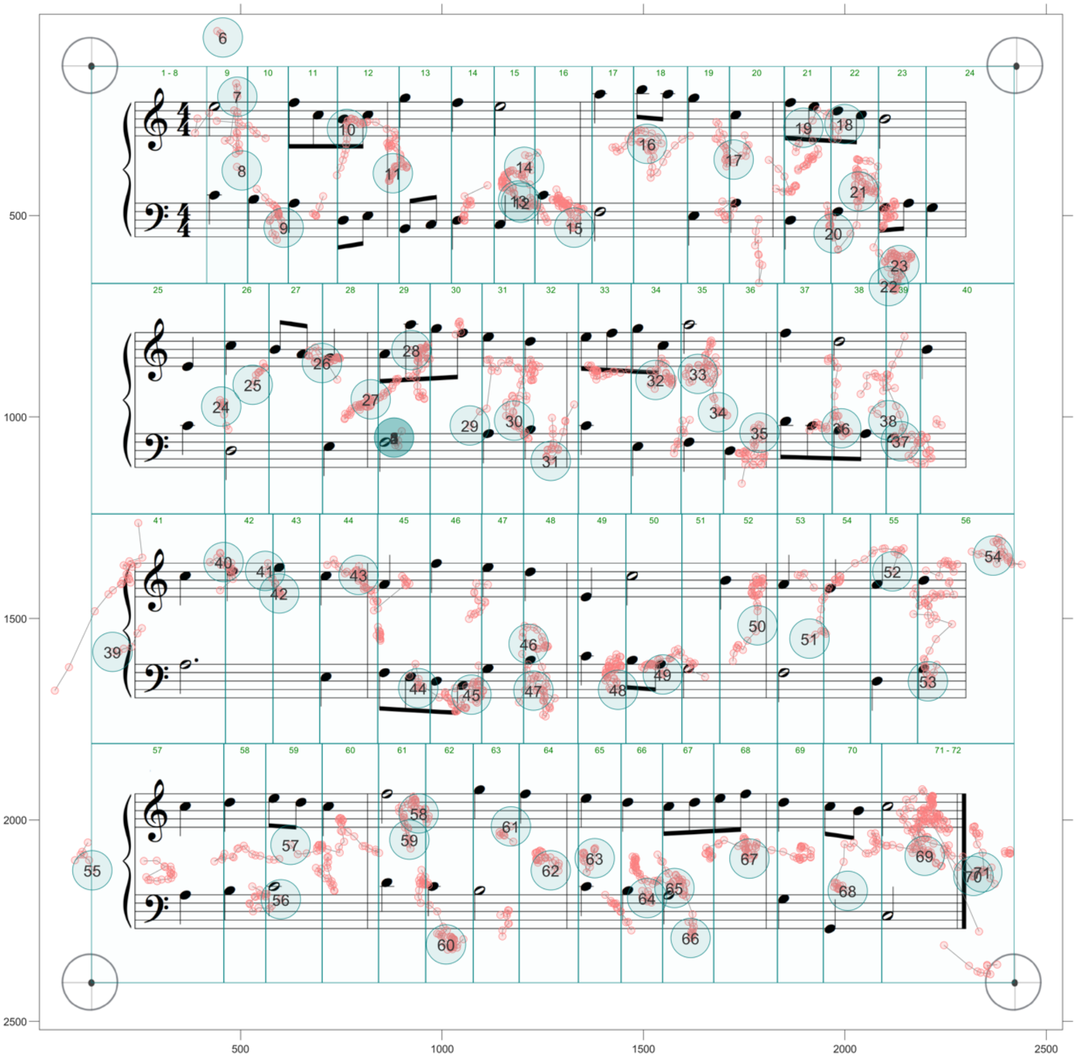

Supplementary Fig. S1 online. Visualization of the EHS numbers indicating the EHS order represented as a beat index.

Supplementary tables

Supplementary Table S1. A summary of the EHS literature.

Paper	Year	Eye Tracker	EHS in Note	EHS in Time	EHS in Beat	Participant Type	N	Playing Tempo	Materials
Weaver[10]	1943	O	1.9-3.1 (1.5 for Chord)			Musicians	15	X	Polyphony, Homophony excerpts
Sloboda[11]	1974	X	3.8-6.8			Musicians (various)	10	O	Single melody
Goolsby[12]	1994	O			4	Musicians (skilled and less-skilled)	2	▵	Single melody
Truitt *et al.*[13]	1997	O			1-2	Pianists	8	O (1st) X (2nd)	Single melody
Furneaux and Land[14]	1999	O	2-4	1s		Pianists (novice, intermediate, and professional)	8 (3, 3, 2)	X	Dual-staved music
Gilman and Underwood[15]	2003	O			¾-1	Pianists	40	X	Bach Chorales
Wurtz *et al.*[16]	2009	O	3-6	1s		Violinists	7	X	Single melody (Corelli, Telemann)
Penttinen *et al.*[17]	2015	O		1s	1-2	Pianist (performance and music education majors)	38 (14, 24)	O	Single melody ('Mary Had a Little Lamb')
Rosemann *et al.*[18]	2016	O		1-1.5 s	0.5	Pianists (professional)		O	Bach Flute Sonata
Cara[19]	2018	O	2.71-7.34		1.36-4.71	Pianist (skilled and less-skilled)	22 (11, 11)	X	Ligeti Piano Etude No. 4
Huovinen *et al.*[9]	2018	O	Eye-Time Span (in metrical time): 2.12 (for Experiment 1), 2.93 (for Experiment 2) beats			Musicians (performance and music education majors)	51 (37, 14)	O	Single melody
	Total		1.9-7.34	1-1.5 s	0.5-4.71				

Note. The selection criteria for the EHS studies were as follows: 1) studies measuring the EHS in a sight-reading performance (e.g., reading and playing a musical piece

simultaneously), and 2) studies published in peer-reviewed journals and written in English. In total, 11 publications fulfilled these criteria. ‘Eye Tracker’ indicates whether an eye tracker was used. ‘EHS’ indicates the standard of the EHS measurement. The EHS was calculated in note, beat, and time indices, and each value in previous research is represented in the table. Until 1997, the EHS was measured only in the note and beat indices (the number of notes and beats between fixation and the performance of a note); however, the time index was introduced in 1999 to enable the calculation of the latency between fixation and the execution of a note. Subsequently, most research measured the EHS in two indices (note and time or beat and time). Recently, the concept of eye-time span (ETS) was proposed by Huovinen *et al.*[9], who redefined looking ahead as a metrical distance between fixation and a corresponding point of metrical time at the onset of fixation on the score. The ETS was represented in the beat index; however, the difference between the EHS (beat) and ETS is that the ETS possesses more continuous measurement styles compared to the EHS (beat) in which discrete events (i.e., the number of beats) are counted. ‘Participants’ indicates the number and type of subjects. ‘Materials’ indicates the type of sight-reading materials used in the experiments. ‘Playing Tempo’ indicates whether the participants’ playing tempo was controlled. X and O indicate the inconsistency and consistency, respectively, of the performance tempo among the participants; △ indicates that a metronome was given to the participants before the sight-reading task but was removed during sight-reading (for a review of playing tempo, please see Huovinen *et al.*[9]; Puurtinen *et al.*[20]; Penttinen *et al.*[17]).

Supplementary Table S2. Integrated accuracy, pitch accuracy, and rhythmic accuracy depending on the musical complexity and playing tempo (mean ± SD).

		Slow	Fast
Integrated accuracy	Simple	11.92 ± 3.34	12.44 ± 3.60
	Complex	3.47 ± 1.54	3.50 ± 1.85
Pitch accuracy	Simple	98.66 ± 2.49	96.6 ± 3.46
	Complex	76.67 ± 12.32	60.31 ± 18.58
Rhythmic accuracy	Simple	99.25 ± 1.3	98 ± 1.08
	Complex	90.72 ± 6.02	86.72 ± 7.61

Supplementary Table S3. *F*-values and *P*-values of the integrated accuracy with different musical complexities and playing tempi.

Factors	*F*-value	*P*-value
Complexity	314.86	0.000***
Playing Tempo	2.38	0.461
Complexity × Playing Tempo	1.80	0.823

***$P < .001$.

Supplementary Table S4. EHS with different musical complexities and playing tempi (mean ± SD).

EHS	Complexity	Slow	Fast
EHS (beat)	Simple	1.62 ± 0.78	1.68 ± 0.72
	Complex	1.27 ± 0.76	1.28 ± 0.69
EHS (sec)	Simple	1.10 ± 0.52	1.11 ± 0.52
	Complex	0.83 ± 0.48	0.82 ± 0.38
EHS (note)	Simple	3.33 ± 1.61	3.52 ± 1.55
	Complex	3.77 ± 2.31	3.76 ± 2.02

Supplementary Table S5. *F*-values and *P*-values of the EHS with different musical complexities and playing tempi.

EHS	Factors	*F*-value	*P*-value
EHS (beat)	Complexity	6.39	0.017*
	Playing Tempo	0.06	0.802
	Complexity × Playing Tempo	0.64	0.431
EHS (sec)	Complexity	7.12	0.012*
	Playing Tempo	0.11	0.741
	Complexity × Playing Tempo	0.68	0.416
EHS (note)	Complexity	1.06	0.311
	Playing Tempo	0.14	0.715
	Complexity × Playing Tempo	0.59	0.450

*$P < .001$.

Supplementary Table S6. Correlation coefficients between the EHS (beat, sec, and not) values and performance accuracy (integrated, pitch, and rhythmic accuracy) values: Pearson correlation coefficients for the integrated accuracy (IA); Spearman correlation coefficients for the pitch and rhythmic accuracy (*P*-value).

		M (*SD*)	IA	Pitch	Rhythmic
Overall	EHS (beat)	1.56 (0.92)	0.22 (0.016)*	0.23 (0.010)*	0.22 (0.014)*
(*N* = 124)	EHS (sec)	1.03 (0.60)	0.26 (0.004)**	0.21 (0.022)*	0.21 (0.025)*
	EHS (note)	3.86 (2.34)	-0.05 (0.570)	-0.06 (0.523)	-0.05 (0.597)
Simple	EHS (beat)	1.76 (0.94)	0.22 (0.085)	0.12 (0.367)	-0.1 (0.432)
(*n* = 62)	EHS (sec)	0.987 (0.50)	0.31 (0.015)*	0.06 (0.664)	-0.15 (0.248)
	EHS (note)	3.68 (1.93)	0.23 (0.078)	0.11 (0.396)	-0.12 (0.372)
Complex	EHS (beat)	1.37 (0.86)	-0.24 (0.057)	-0.19 (0.133)	0.08 (0.522)
(*n* = 62)	EHS (sec)	0.90 (0.58)	-0.16 (0.207)	-0.19 (0.146)	0.09 (0.487)
	EHS (note)	4.06 (2.59)	-0.25 (0.049)*	-0.18 (0.171)	0.08 (0.515)

***$P < 0.001$, **$P < 0.01$, *$P < 0.05$.

Supplementary Table S7. Correlations between the EHS and integrated accuracy in the high and low groups.

Group	EHS	Integrated accuracy [Spearman's rho (*P*-value)]			
		Simple-Slow	Simple-Fast	Complex-Slow	Complex-Fast
High (*n* = 10)	EHS (beat)	0.75 (0.013)*	0.10 (0.777)	0.08 (0.829)	-0.78 (0.008)**
	EHS (sec)	0.75 (0.013)*	0.44 (0.20)	0.07 (0.855)	-0.69 (0.029)*
	EHS (note)	0.75 (0.013)*	0.10 (0.777)	0.08 (0.829)	-0.76 (0.011)*
Low (*n* = 10)	EHS (beat)	0.10 (0.777)	-0.72 (0.019)*	-0.16 (0.651)	-0.61 (0.060)
	EHS (sec)	-0.06 (0.881)	-0.38 (0.276)	-0.07 (0.855)	-0.65 (0.043)*
	EHS (note)	0.10 (0.777)	-0.71 (0.022)*	--0.18 (0.627)	-0.58 (0.082)

$^{**}P < 0.01$, $^{*}P < 0.05$.

Supplementary Table S8. Quantitative schema of the sight-reading materials.

	Sight-reading materials		
	Simple	Complex	*t* (*P*-value)
**Notes per beat**	2.03 ± 0.71	2.97 ± 0.81	-9.83 (0.000) ***
**Accidentals (# or ♭)**	0	84	
**Half notes**	19	Null	
**Simultaneous occurrence of two voices per beat**	0.70 ± 0.48	0.96 ± 0.59	-3.83 (0.000) ***
**Syncopated notes**	0	28 - 30	
**Key**	C Major		
**Meter**	4/4		
**Length**	16 measures		
**Tempo (BPM)**	80 for the slow tempo; 104 for the fast tempo		

***$P < .001$.

Note. Significantly more notes per beat were found in the complex pieces (mean ± SD = 2.97 ± 0.81) than in the simple pieces (mean ± SD = 2.97 ± 0.81) after conducting an independent *t*-test [$t(254) = -9.83$ ($P < .001$)]. Significantly more simultaneous occurrences of two voices per beat were found in the complex pieces (mean ± SD = 0.96 ± 0.59) than in the simple pieces (mean ± SD = 0.70 ± 0.48) after conducting an independent *t*-test [$t(254) = -3.83$ ($P < .001$)]. The complex pieces were more difficult than the simple pieces in terms of note duration because the complex pieces had more syncopation (28 – 30), whereas the simple pieces did not have any syncopation.

References

1 Olsen, A. The Tobii I-VT fixation filter. http://www.tobii.com/no/eye-trackingresearch/global/library/white-papers/the-tobii-i-vt-fixation-filter/ (2012).

2 Müller, M. Dynamic time warping. In *Information retrieval for music and motion* (ed Müller, M.) 69-84 (Springer, 2007).

3 Soulez, F., Rodet, X. & Schwarz, D. Improving polyphonic and polyinstrumental music to score alignment. In *4th international conference on music information retrieval* (ISMIR, 2003).

4 Molina, E., Barbancho, I., G´omez, E., Barbancho, A. M. & Tard´on, L. J. Fundamental frequency alignment vs. note-based melodic similarity for singing voice assessment. In *Proceedings of 2013 IEEE international conference on acoustics, speech, and signal processing* (ICASS, 2013).

5 Bozjurt, B., Baydal, O. & Yüret, D. A dataset and baseline system for singing voice assessment. In *Proceedings of the 13th international symposium on computer music multidisciplinary research* (CMMR, 2017).

6 Pan, J. *et al.* An audio based piano performance evaluation method using deep neural network based acoustic modeling. In *Proceedings of INTERSPEECH 2017* (2017).

7 Vidwans, A. *et al.* Gururani, S., Wu, C., Subramanian, V., Swaminathan, R. V. & Lerch, A. Objective descriptors for the assessment of student music performances. In *Proceedings of 2017 AES international conference on semantic audio, audio engineering society* (2017).

8 Nakamura, E., Yoshii, K. & Katayose, H. Performance error detection and post-processing for fast and accurate symbolic music alignment. *In Proceedings of 18th international society for music information retrieval conference*, 347-353 (2017).

9 Huovinen, E., Ylitalo, A.-K. & Puurtinen, M. Early attraction in temporally controlled sight reading of music. *J. Eye Mov. Res.* **11** (2018).

10 Weaver, H. E. A survey of visual processes in reading differently constructed musical selections. *Psychol. Monogr.* **55,** 1-30 (1943).

11 Sloboda, J. The eye-hand span-an approach to the study of sight reading. *Psychol. Music* **2,** 4-10 (1974).

12 Goolsby, T. W. Eye movement in music reading: effects of reading ability, notational complexity, and encounters. *Music Percept.* **12,** 77-96 (1994).

13 Truitt, F. E., Clifton, C., Pollatsek, A. & Rayner, K. The perceptual span and the eye-hand span in sight reading music. *Vis. Cogn.* **4,** 143-161 (1997).

14 Furneaux, S. & Land, M. F. The effects of skill on the eye-hand span during musical sight-reading. *Proc. Biol. Sci.* **266,** 2435-2440 (1999).

15 Gilman, E. & Underwood, G. Restricting the field of view to investigate the perceptual spans of pianists. *Vis. Cogn.* **10,** 201-232 (2003).

16 Wurtz, P., Mueri, R. M. & Wiesendanger, M. Sight-reading of violinists: eye movements anticipate the musical flow. *Exp. Brain Res.* **194,** 445-450 (2009).

17 Penttinen, M., Huovinen, E. & Ylitalo, A.-K. Reading ahead: adult music students' eye movements in temporally controlled performances of a children's song. *Int. J. Music Educ.* **33,** 36-50 (2015).

18 Rosemann, S., Altenmüller, E. & Fahle, M. The art of sight-reading: influence of practice, playing tempo, complexity and cognitive skills on the eye–hand span in pianists. *Psychol. Music* **44,** 658-673 (2016).

19 Cara, M. A. Anticipation awareness and visual monitoring in reading contemporary music. *Mu sic Sci.* **22,** 322-343 (2018).

20 Puurtinen, M. Eye on music reading: A methodological review of studies from 1994 to 2017. *J. Eye Mov. Res.* **11,** 1-16 (2018).
